# Supplementary material for: Phylogenetic and functional characterization of Asgard primases
Source: Mol Biol Evol. 2025 Dec 23;43(1):msaf330. doi: 10.1093/molbev/msaf330 (PMC12770985; doi:10.1093/molbev/msaf330)
Supplement: msaf330_Supplementary_Data [file msaf330_supplementary_data.pdf]

Supplementary materials

Phylogenetic and functional characterization of Asgard primases

Zhimeng Li[1,2,3#], Yang Liu[2#], Li Huang[1,3*] and Meng Li[2*]

[1]Southern Marine Science and Engineering Guangdong Laboratory (Guangzhou), No. 1119 Haibin Road, Nansha District, Guangzhou 511458, China

[2]Archaeal Biology Center, Synthetic Biology Research Center, Shenzhen Key Laboratory of Marine Microbiome Engineering, Key Laboratory of Marine Microbiome Engineering of Guangdong Higher Education Institutes, Institute for Advanced Study, Shenzhen University, 3688 Nanhai Avenue, Shenzhen 518060, China.

[3]State Key Laboratory of Microbial Resources, Institute of Microbiology, Chinese Academy of Sciences, No. 1 West Beichen Road, Chaoyang District, Beijing 100101, China.

[#] Co-first Author

[*] Corresponding authors. Meng Li, Institute for Advanced Study, Shenzhen University, 3688 Nanhai Avenue, Shenzhen 518060, China. Email: limeng848@szu.edu.cn; Li Huang, Southern Marine Science and Engineering Guangdong Laboratory (Guangzhou), Nansha, Guangzhou 511458, China. Email: huang_li@gmlab.ac.cn.

This file includes:

Supplementary Figure 1-10

Supplementary Table 1-6

A

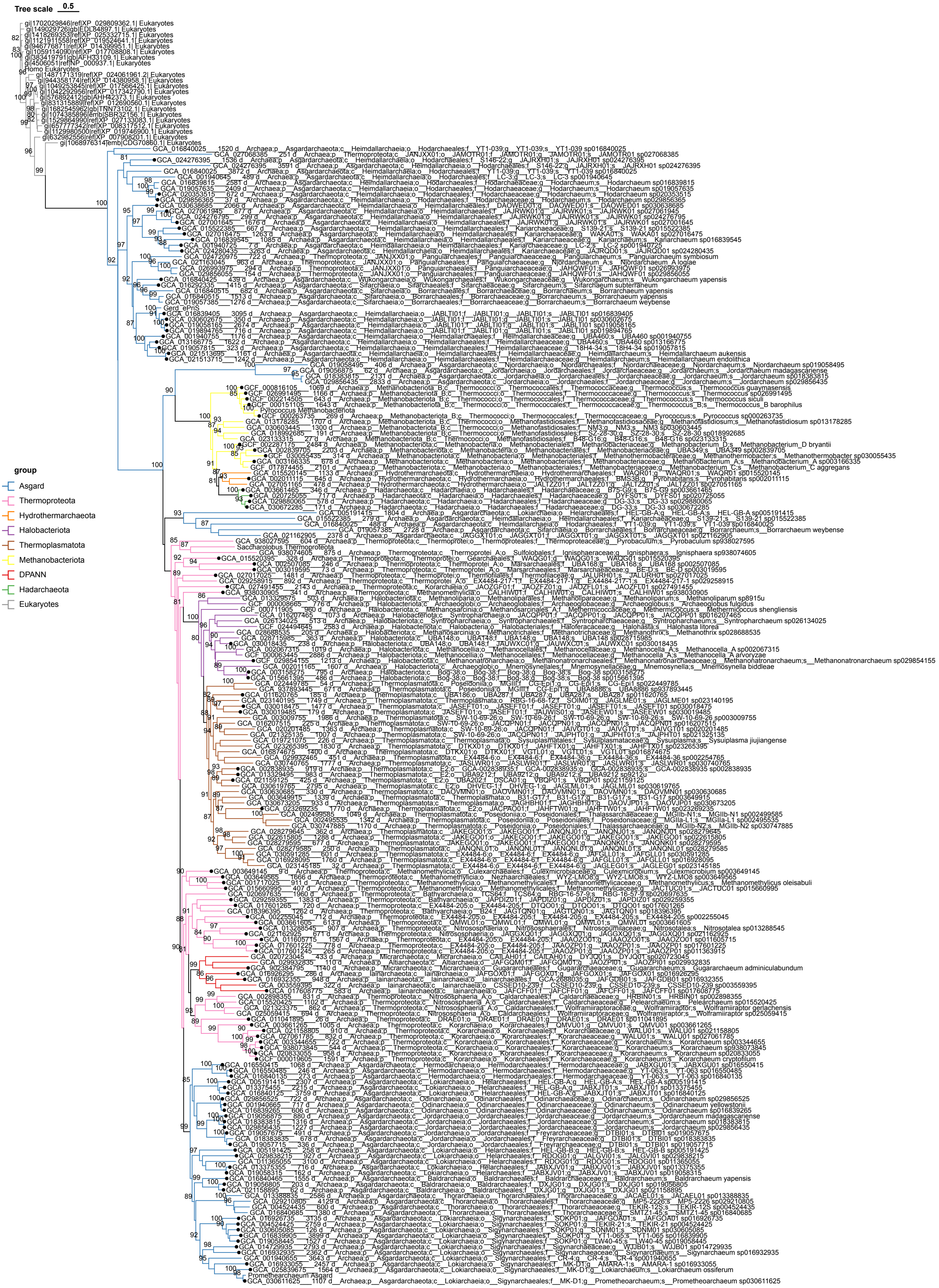
Tree scale
0.5
group
Asgard
Thermoproteota
Hydrothermarchaeota
Halobacteriota
Thermoplasmatota
Methanobacteriota
DPANN
Hadarchaeota
Eukaryotes

B

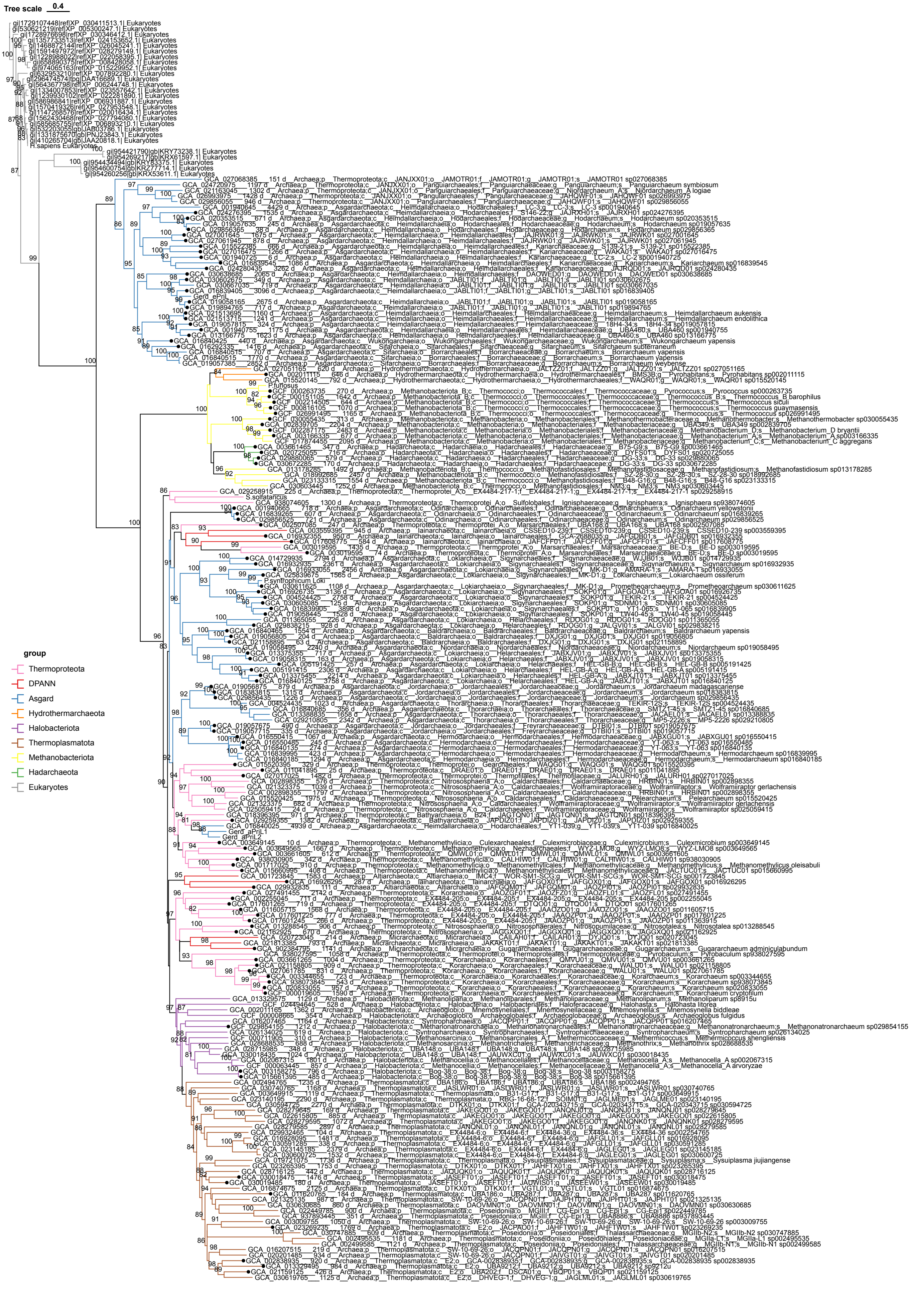

Tree scale 0.4
group
Thermoproteota
DPANN
Asgard
Hydrothermarchaeota
Halobacteriota
Thermoplasmatota
Methanobacteriota
Hadarchaeota
Eukaryotes

Supplementary Figure 1. Phylogenetic trees of primases. (A) Maximum likelihood phylogenetic tree of PriS. **(B)** Maximum likelihood phylogenetic tree of PriL. Detailed information of the proteins used to construct the phylogenetic tree is displayed in the figures. Solid dots indicate that genes encoding PriS and PriL are adjacently located on the genome.

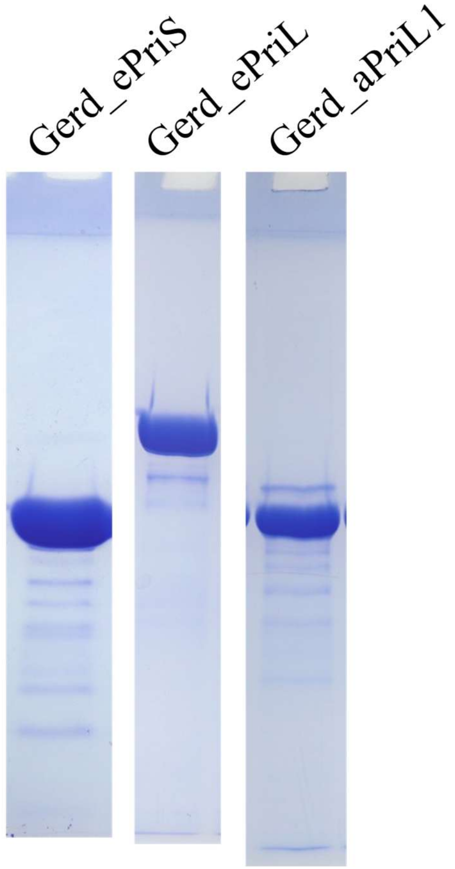

Supplementary Figure 2. Purification of B18_G1 primase subunits. Analysis of purified proteins by 12% SDS-PAGE.

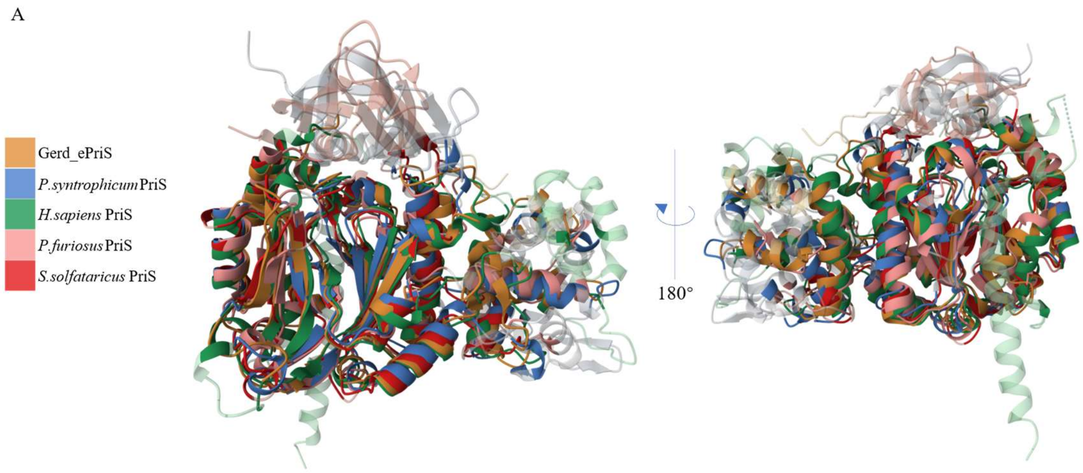

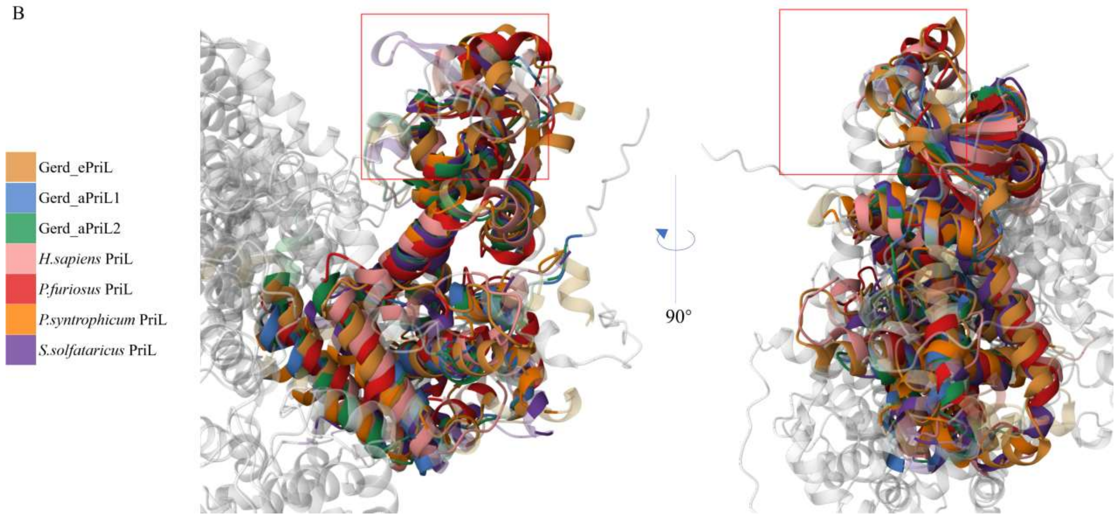

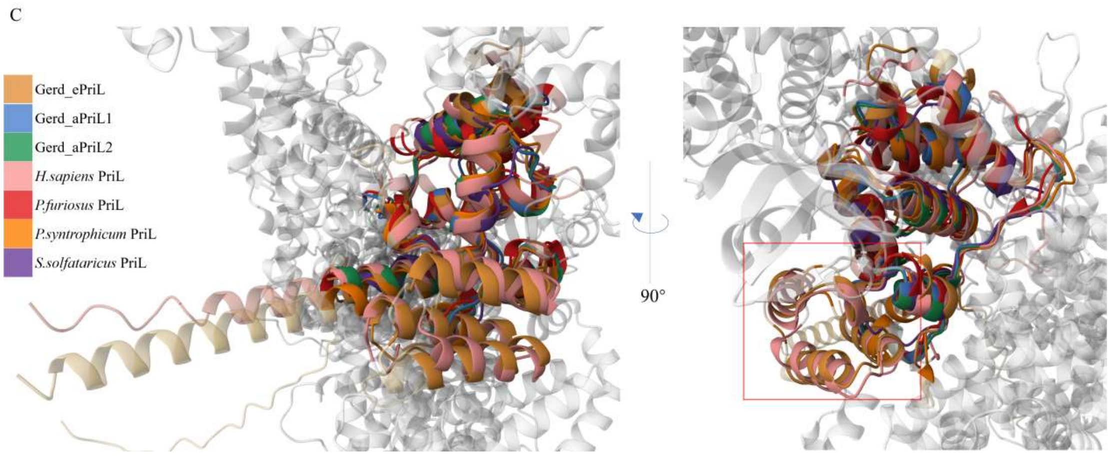

Supplementary Figure 3. Structural comparison of selected archaeal and eukaryotic

primases. (A) PriS. **(B)** The N-terminal domain of PriL. **(C)** The C-terminal domain of PriL. The structures of primases from *Candidatus* Gerdarchaeota archaeon B18_G1 (Gerd) and *Promethearchaeum syntrophicum* (*P. syntrophicum*) were predicted with AlphaFold, and those from human (*H. sapiens*; PDB: 4MHQ & 5EXR), *Saccharolobus solfataricus* (*S. solfataricus*; PDB: 5OF3), and *Pyrococcus furiosus* (*P. furiosus*; PDB: 1g71 & 9F28) were retrieved from RCSB PDB. The C-terminal part of PriL (Table S2) was predicted with AlphaFold3. Structurally distinct regions in PriLs of different origins are boxed. The colors corresponding to the different structures are indicated. The N-terminal and C-terminal domains of PriL were aligned in separate structural comparisons. The gray-white portion shows the C-terminal domain **(B)** or the N-terminal domain **(C)** of PriL. Structural alignments were performed using the Pairwise Structure Alignment tool on PCSB PDB with TM-align method.

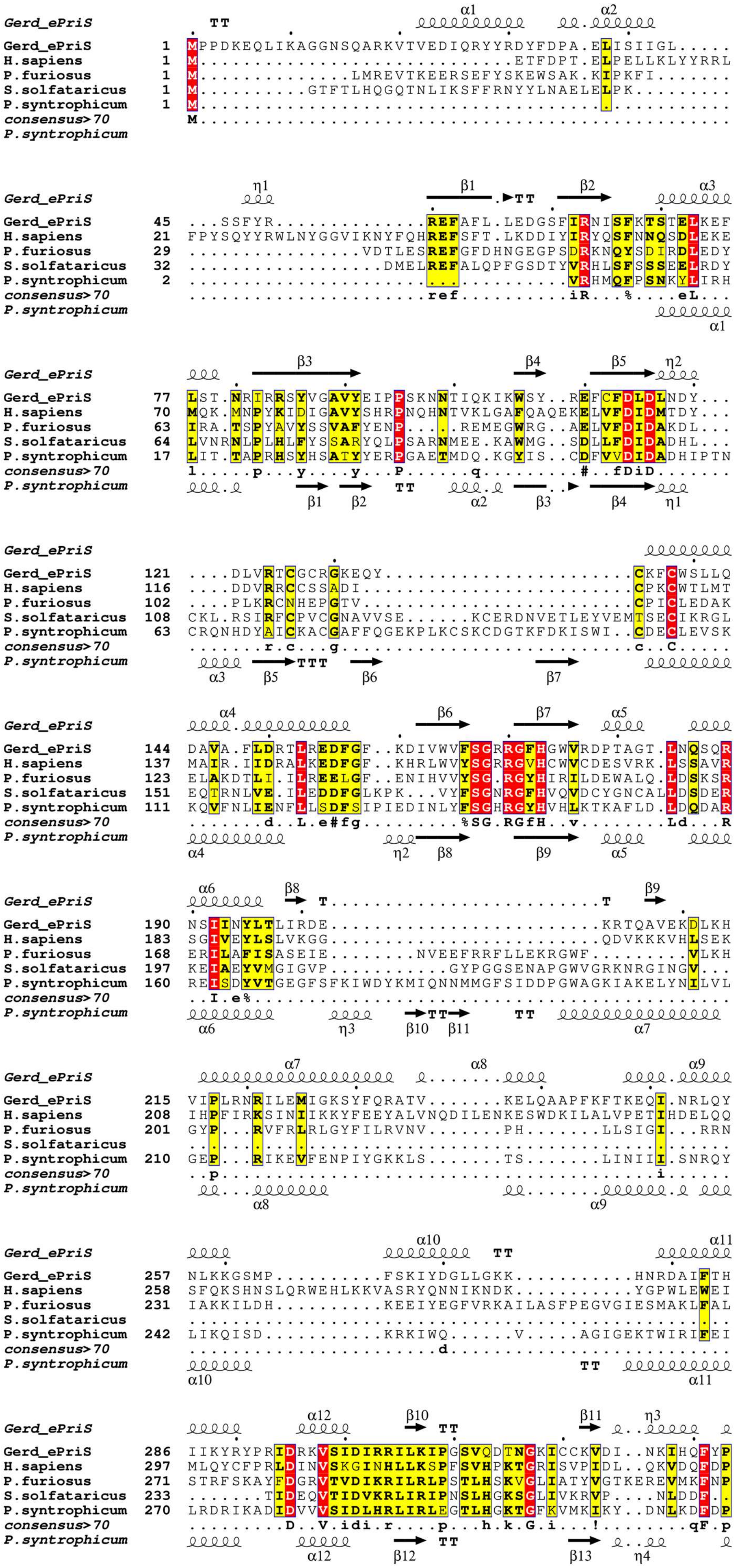
Gerd_ePriS 1 MPPDKEQLIKAGGNSQARKVTVEDIQRYYRDYFDPA.ELISIIGL.....
H.sapiens 1 M...........................ETFDPT.ELPELLKLYYRRL
P.furiosus 1 M............LMREVTKEERSEFYSKEWSAK.KIPKFI.......
S.solfataricus 1 M.........GTFTLHQGQTNLIKSFFRNYYLNAELELPK........
P.syntrophicum 1 M
consensus>70 M
Gerd_ePriS 45 ...SSFYR...............REFAFL.LEDGSFIRNISFKTSTELKEF
H.sapiens 21 FPYSQYYRWLNYGGVIKNYFQHREFSFT.LKDDIYIRYQSFNNQSDLEKE
P.furiosus 29 ...............VDTLESREFGFDHNGEGPSDRKNQYSDIRDLEDY
S.solfataricus 32 .................DMELREFALQPFGSDTYVRHLSFSSSEELRDY
P.syntrophicum 2 VRHMQFPSNKYLIRH
consensus>70 ref iR % eL
Gerd_ePriS 77 LST.NRIRRSYVGAVYEIPPSKNNTIQKIKWSY..REFCFDLDLNDY...
H.sapiens 70 MQK.MNPYKIDIGAVYSHRPNQHNTVKLGAFQAQEKELVFDIDMTDY...
P.furiosus 63 IRA.TSPYAVYSSVAFYENP.....REMEGWRG..AELVFDIDAKDL...
S.solfataricus 64 LVNRNLPLHLFYSSARYQLPSARNMEE.KAWMG..SDLLFDIDADHL...
P.syntrophicum 17 LIT.TAPRHSYHSATYYERPGAETMDQ.KGYIS..CDFVVDIDADHIPTN
consensus>70 l p y y P q # fDiD
Gerd_ePriS 121 ....DLVRTCGCRGKEQY CKFCWSLLQ
H.sapiens 116 ....DDVRRCCSSADI CPKCWTLMT
P.furiosus 102 ....PLKRCNHEPGTV CPICLEDAK
S.solfataricus 108 CKL.RSIRFCPVCGNAVVSE......KCERDNVETLEYVEMTSECIKRGL
P.syntrophicum 63 CRQNHDYAICKACGAFFQGEKPLKCSKCDGTKFDKISWI..CDECLEVSK
consensus>70 r c g c C
Gerd_ePriS 144 DAVA.FLDRTLREDFGF..KDIVWVFSGRRGFHGWVRDPTAGT.LNQSQR
H.sapiens 137 MAIR.IIDRALKEDFGF..KHRLWVYSGRRGVHCWVCDESVRK.LSSAVR
P.furiosus 123 ELAKDTLI.ILREELGF..ENIHVVYSG.RGYHIRILDEWALQ.LDSKSR
S.solfataricus 151 EQTRNLVE.ILEDDFGLKPK...VYFSGNRGFHVQVDCYGNCALLDSDER
P.syntrophicum 111 KQVFNLIENFLLSDFSIPIEDINLYFSGHRGYHVHLKTKAFLD.LDQDAR
consensus>70 d L e#fg %SG.RGfH v Ld R
Gerd_ePriS 190 NSIINYLTLIRDE KRTQAVEKDLKH
H.sapiens 183 SGIVEYLSLVKGG QDVKKVHLSEK
P.furiosus 168 ERILAFISASEIE........NVEEFRRFLLEKRGWF.........VLKH
S.solfataricus 197 KEIAEYVMGIGVP.........GYPGGSENAPGWVGRKNRGINGV...
P.syntrophicum 160 REISDYVTGEGFSFKIWDYKMIQNNMMGFSIDDPGWAGKIAKELYNILVL
consensus>70 I.e%
Gerd_ePriS 215 VIPLRNRILEMIGKSYFQRATV.......KELQAAPFKFTKEQI.NRLQY
H.sapiens 208 IHPFIRKSINIIKKYFEEYALVNQDILENKESWDKILALVPETIHDELQQ
P.furiosus 201 GYP...RVFRLRLGYFILRVNV.......PH.......LLSIGI...RRN
S.solfataricus
P.syntrophicum 210 GEP...RIKEVFENPIYGKKLS.......TS.......LINIII.SNRQY
consensus>70 p i
Gerd_ePriS 257 NLKKGSMP..........FSKIYDGLLGKK...........HNRDAIFTH
H.sapiens 258 SFQKSHNSLQRWEHLKKVASRYQNNIKNDK...........YGPWLEWEI
P.furiosus 231 IAKKILDH..........KEEIYEGFVRKAILASFPEGVGIESMAKLFAL
S.solfataricus
P.syntrophicum 242 LIKQISD...........KRKIWQ......V.....AGIGEKTWIRIFEI
consensus>70 d
Gerd_ePriS 286 IIKYRYPRIDRKVSIDIRRILKIPGSVQDTNGKICCKVDI..NKIHQFYP
H.sapiens 297 MLQYCFPRLDINVSKGINHLLKSPFSVHPKTGRISVPIDL..QKVDQFDP
P.furiosus 271 STRFSKAYFDGRVTVDIKRILRLPSTLHSKVGLIATYVGTKEREVMKFNP
S.solfataricus 233 .......TIDEQVTIDVKRLIRIPNSLHGKSGLIVKRVP....NLDDF..
P.syntrophicum 270 LRDRIKADIDVVSIDLHRLIRLEGTLHGKTGFKVMKIKY..DNLKDFDP
consensus>70 D V idi r p h k G i ! qF p

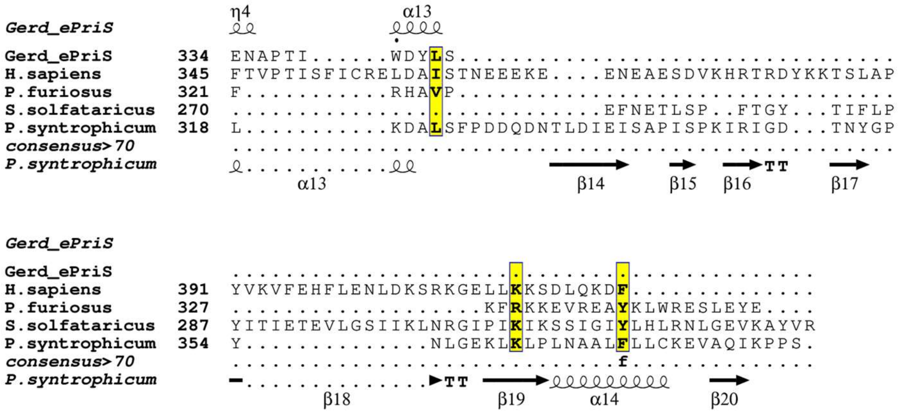

Supplementary Figure 4. Secondary structure comparison of PriS. The top panel shows the secondary structure diagram of Gerd_ePriS (squiggles for helices, arrows for β-strands and TT letters for turns), and the bottom panel displays that of PriS from *Candidatus Prometheoarchaeum syntrophicum* strain MK-D1. Secondary structure alignment was performed with ESPript 3.0. Identical and similar residues are boxed in red and yellow, respectively.

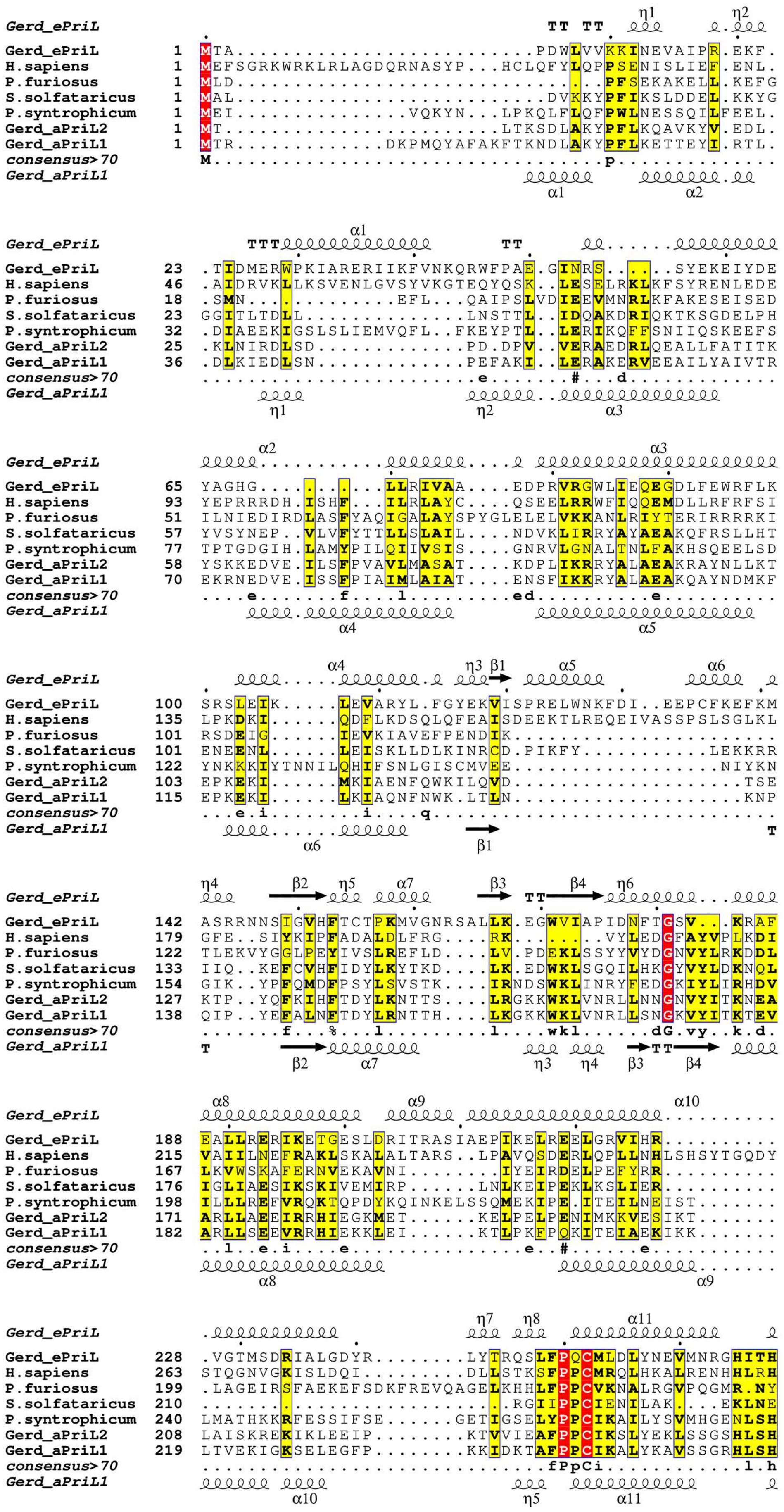

Gerd_ePriL TT TT η1 η2
Gerd_ePriL 1 MTA.........................PDWLVVKKINEVAIPR.EKF.
H.sapiens 1 MEFSGRKWRKLRLAGDQRNASYP...HCLQFYLQPPSENISLIEF.ENL.
P.furiosus 1 MLD..........................PFSEKAKELL.KEFG
S.solfataricus 1 MAL........................DVKKYPFIKSLDDEL.KKYG
P.syntrophicum 1 MEI..............VQKYN...LPKQLFLQFPWLNESSQILFEEL.
Gerd_aPriL2 1 MT.....................LTKSDLAKYPFLKQAVKYV.EDL.
Gerd_aPriL1 1 MTR............DKPMQYAFAKFTKNDLAKYPFLKETTEYI.RTL.
consensus>70 M..........................p..........
Gerd_aPriL1 α1 α2
Gerd_ePriL TTT α1 TT
Gerd_ePriL 23 .TIDMERWPKIARERIIKFVNKQRWFPAE.GINRS.......SYEKEIYDE
H.sapiens 46 .AIDRVKLLKSVENLGVSYVKGTEQYQSK..LESELRKLKFSYRENLEDE
P.furiosus 18 .SMN.............EFL...QAIPSLVDIEEVMNRLKFAKESEISED
S.solfataricus 23 GGITLTDLL.............LNSTTL..IDQAKDRIQKTKSGDELPH
P.syntrophicum 32 .DIAEEKIGSLSLIEMVQFL..FKEYPTL..LERIKQFFSNIIQSKEEFS
Gerd_aPriL2 25 .KLNIRDLSD............PD.DPV..VERAEDRLQEALLFATITK
Gerd_aPriL1 36 .DLKIEDLSN............PEFAKI..LERAKERVEEAILYAIVTR
consensus>70 ....e.......#...d..........
Gerd_aPriL1 η1 η2 α3
Gerd_ePriL α2 α3
Gerd_ePriL 65 YAGHG.........LLRIVAA....EDPRVRGWLIEQEGDLFEWRFLK
H.sapiens 93 YEPRRRDH.ISHF...ILRLAYC....QSEELRRWFIQQEMDLLRFRFSI
P.furiosus 51 ILNIEDIRDLASFYAQIGALAYSPYGLELELVKKANLRIYTERIRRRRKI
S.solfataricus 57 YVSYNEP..VLVFYTTLLSLAIL....NDVKLIRRYAYAEAKQFRSLLHT
P.syntrophicum 77 TPTGDGIH.LAMYPILQIIVSIS....GNRVLGNALTNLFAKHSQEELSD
Gerd_aPriL2 58 YSKKEDVE.ILSFPVAVLMASAT....KDPLIKRRYALAEAKRAYNLLKT
Gerd_aPriL1 70 EKRNEDVE.ISSFPIAIMLAIAT....ENSFIKKRYALAEAKQAYNDMKF
consensus>70 ....e.......f....l.........ed.........e.........
Gerd_aPriL1 α4 α5
Gerd_ePriL α4 η3 β1 α5 α6
Gerd_ePriL 100 SRSLEIK......LEVARYL.FGYEKVISPRELWNKFDI..EEPCFKEFKM
H.sapiens 135 LPKDKI......QDFLKDSQLQFEAISDEEKTLREQEIVASSPSLSGLKL
P.furiosus 101 RSDEIG......IEVKIAVEFPENDIK.......................
S.solfataricus 101 ENEENL......LEISKLLDLKINRCD.PIKFY...........LEKKRR
P.syntrophicum 122 YNKKKIYTNNILQHIFSNLGISCMVEE.................NIYKN
Gerd_aPriL2 103 EPKEKI......MKIAENFQWKILQVD...................TSE
Gerd_aPriL1 115 EPKEKI......LKIAQNFNWK.LTLN...................KNP
consensus>70 ...e.i.........i....q..........................
Gerd_aPriL1 α6 β1 T
Gerd_ePriL η4 β2 η5 α7 β3 TT β4 η6
Gerd_ePriL 142 ASRRNNSIGVHFTCTPKMVGNRSALLK.EGWVIAPIDNFTGSV...KRAF
H.sapiens 179 GFE..SIYKIPFADALDLFRG....RK.........VYLEDGFAYVPLKDI
P.furiosus 122 TLEKVYGGLPEYIVSLREFLD....LV.PDEKLSSYYVYDGNVYLRKDDL
S.solfataricus 133 IIQ..KEFCVHFIDYLKYTKD....LK.EDWKLSGQILHKGYVYLDKNQL
P.syntrophicum 154 GIK..YPFQMDFPSYLSVSTK....IRNDSWKLINRYFEDGKIYLIRHDV
Gerd_aPriL2 127 KTP..YQFKIHFTDYLKNTTS....LRGKKWKLVNRLLNNGNVYITKNEA
Gerd_aPriL1 138 QIP..YEFALNFTDYLRNTTH....LKGKKWKLVNRLLSNGKVYITKTEV
consensus>70 ....f...%...l.........l....wkl......dG.vy..k.d.
Gerd_aPriL1 T β2 α7 η3 η4 β3 TT β4
Gerd_ePriL α8 α9 α10
Gerd_ePriL 188 EALLRERIKETGESLDRITRASIAEPIKELREELGRVIHR..........
H.sapiens 215 VAIILNEFRAKLSKALALTARS..LPAVQSDERLQPLLNHLSHSYTGQDY
P.furiosus 167 LKVWSKAFERNVEKAVNI........IYEIRDELPEFYRR..........
S.solfataricus 176 IGLIAESIKSKIVEMIRP......LNLKEIPEKLKSLIER..........
P.syntrophicum 198 ILLLREFVRQKTQPDYKQINKELSSQMEKIPE.ITEILNEIST.......
Gerd_aPriL2 171 ARLLAEEIRRHIEGKMET......KELPELPENIMKKVESIKT.......
Gerd_aPriL1 182 ARLLSEEVRRHIEKKLEI......KTLPKFPQKITEIAEKIKK.......
consensus>70 ..l..e.i....e.................e..#......e.........
Gerd_aPriL1 α8 α9
Gerd_ePriL η7 η8 α11
Gerd_ePriL 228 .VGTMSDRIALGDYR........LYTRQSLFPQCMLDLYNEVMNRGHITH
H.sapiens 263 STQGNVGKISLDQI.........DLLSTKSFPPCMRQLHKALRENHHLRH
P.furiosus 199 .LAGEIRSFAEKEFSDKFREVQAGELKHHLFPPCVKNALRGVPQGMR.NY
S.solfataricus 210 ........................RGIIPPCIENILAK....EKLNE
P.syntrophicum 240 LMATHKKRFESSIFSE......GETIGSELYPPCIKAILYSVMHGENLSH
Gerd_aPriL2 208 LAISKREKIKLEEIP........KTVVIEAFPPCIKSLYEKLSSGSHLSH
Gerd_aPriL1 219 LTVEKIGKSELEGFP........KKIDKTAFPPCIKALYKAVSSGRHLSH
consensus>70 ....fPpCi............l.h
Gerd_aPriL1 α10 η5 α11

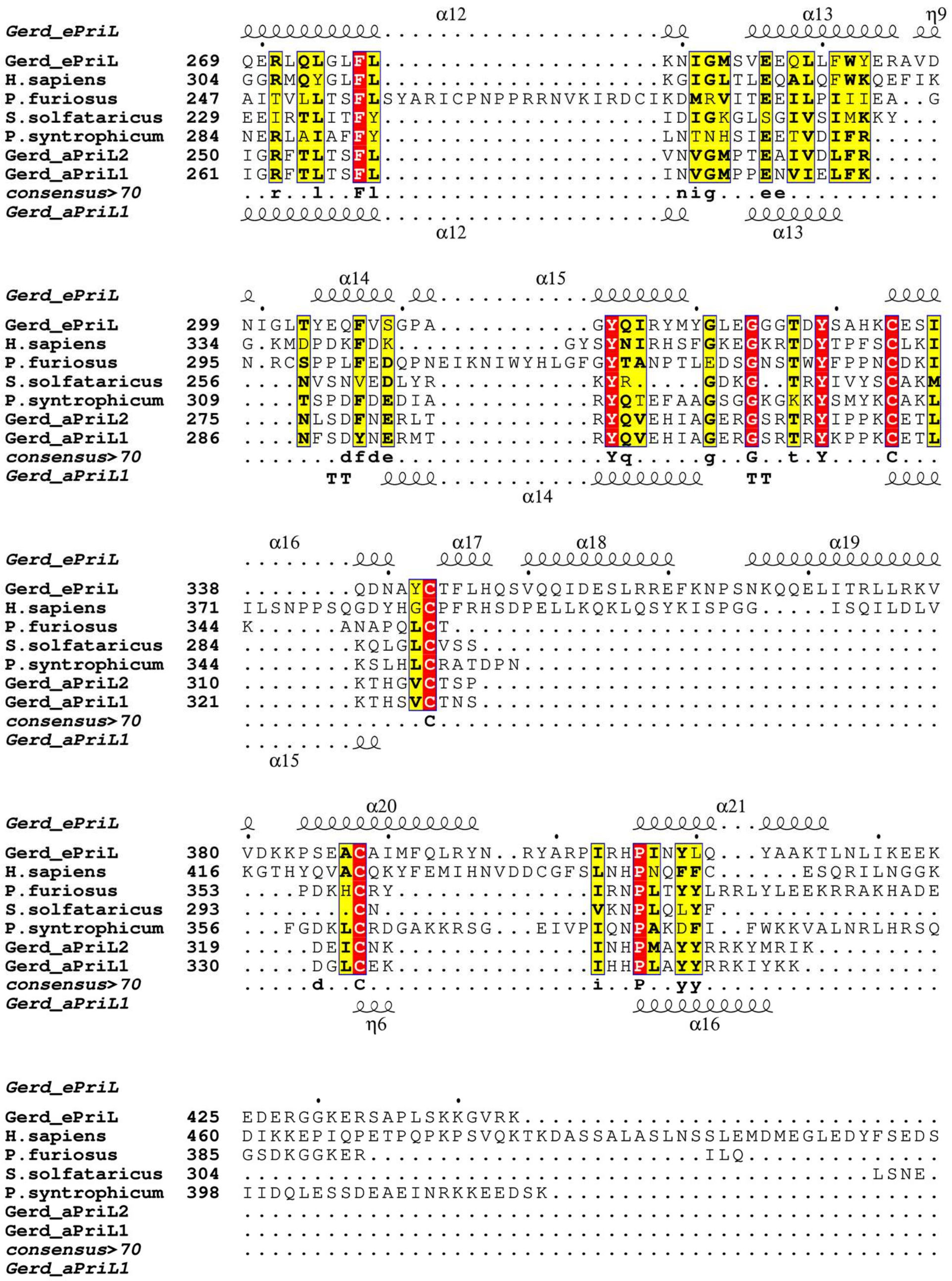

Supplementary Figure 5. Secondary structure comparison of PriL. The top panel shows the secondary structure diagram of Gerd_ePriL (squiggles for helices, arrows for β-strands and TT letters for turns), and the bottom panel displays that of Gerd_aPriL1. Secondary structure alignment was performed with ESPript 3.0. Identical and similar residues are boxed in red and yellow, respectively.

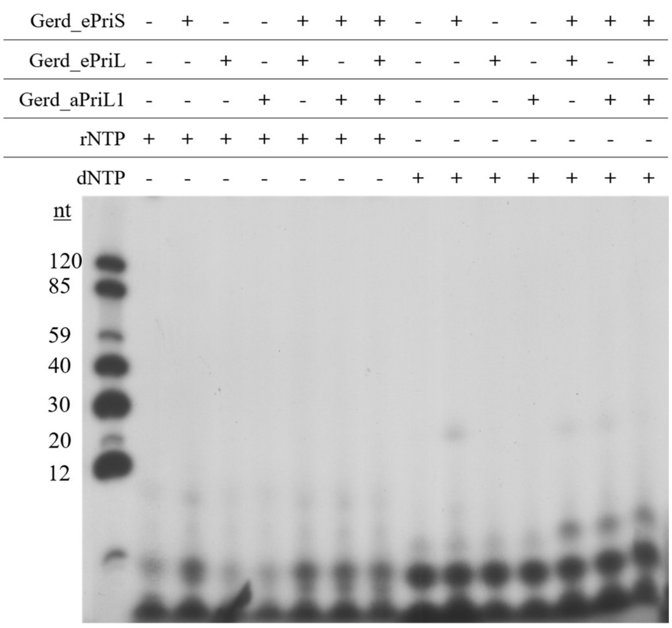

Supplementary Figure 6. Primer synthesis by B18_G1 primase. Reactions were performed by incubating single-subunit enzyme or multi-subunit complexes (1.5 μM) with DNA M13mp18 ssDNA (230 ng) and 10 μM dNTPs (1 μCi [α-^{32}P]dATP) or 10 μM rNTPs (1 μCi [α-^{32}P]dATP) in the standard assay mixture for 30 min at 55ºC. Reactions were stopped by the addition of SDS (0.8%) and protease K (1.6 mg/ml). The products were extracted with phenol/chloroform/isoamyl alcohol (25:24:1), precipitated with ethanol, and analyzed on 20% polyacrylamide gel (19:1) containing 8 M urea. The gel was exposed to X-ray film.

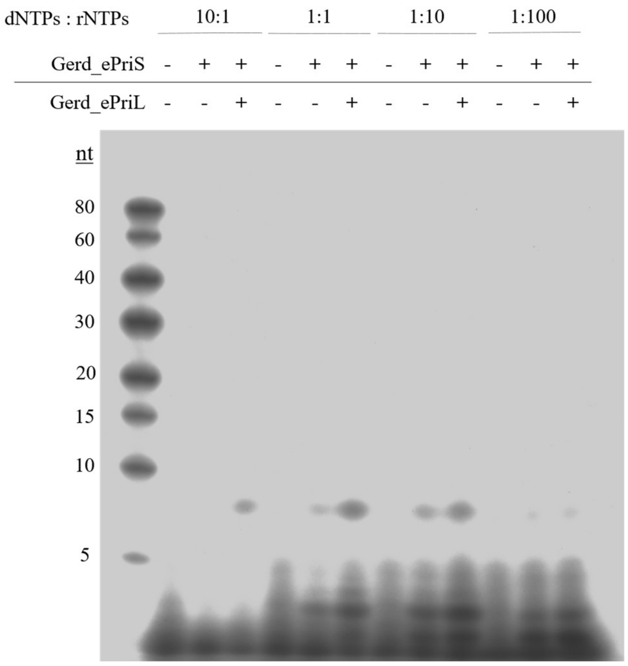

Supplementary Figure 7. Primer synthesis by B18_G1 primase at various dNTPs/rNTPs ratios. Reactions were performed by incubating Gerd_ePriS or Gerd_ePriS-ePriL (1.5 μM) with 230 ng M13mp18 ssDNA and different types of substrates in the standard assay mixture for 30 min at 55°C. The concentrations of dNTPs were 10 μM (1 μCi [α-32P]dATP). The concentrations of rNTPs were added according to the molar ratios shown at the top of the figure. Reactions were stopped by the addition of SDS (0.8%) and protease K (1.6 mg/ml). The products were extracted with phenol/chloroform/isoamyl alcohol (25:24:1), precipitated with ethanol, and analyzed on 25% polyacrylamide gel (19:1) containing 6 M urea. The gel was exposed to X-ray film.

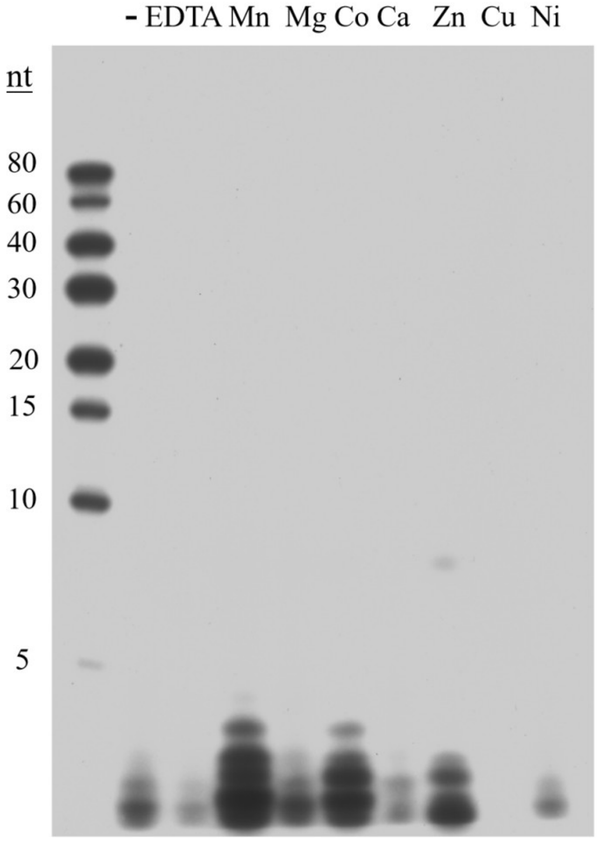

Supplementary Figure 8. Effect of divalent cations on primer synthesis by Gerd_ePriSL. The reaction mixture, containing Gerd_ePriS-ePriL (1.5 μM), M13mp18 ssDNA (230ng), 10 μM dNTPs (1 μCi [α-^{32}P]dATP) and 100 μM rNTPs, 50 mM MES-NaOH, pH7.0, 100 μg/ml BSA, and indicated divalent cations, was incubated at 55°C for 30 min. Reactions were stopped by the addition of SDS (0.8%) and protease K (1.6 mg/ml). The products were extracted with phenol/chloroform/isoamyl alcohol (25:24:1), precipitated with ethanol, and analyzed on 25% polyacrylamide gel (19:1) containing 6 M urea. The gel was exposed to X-ray film.

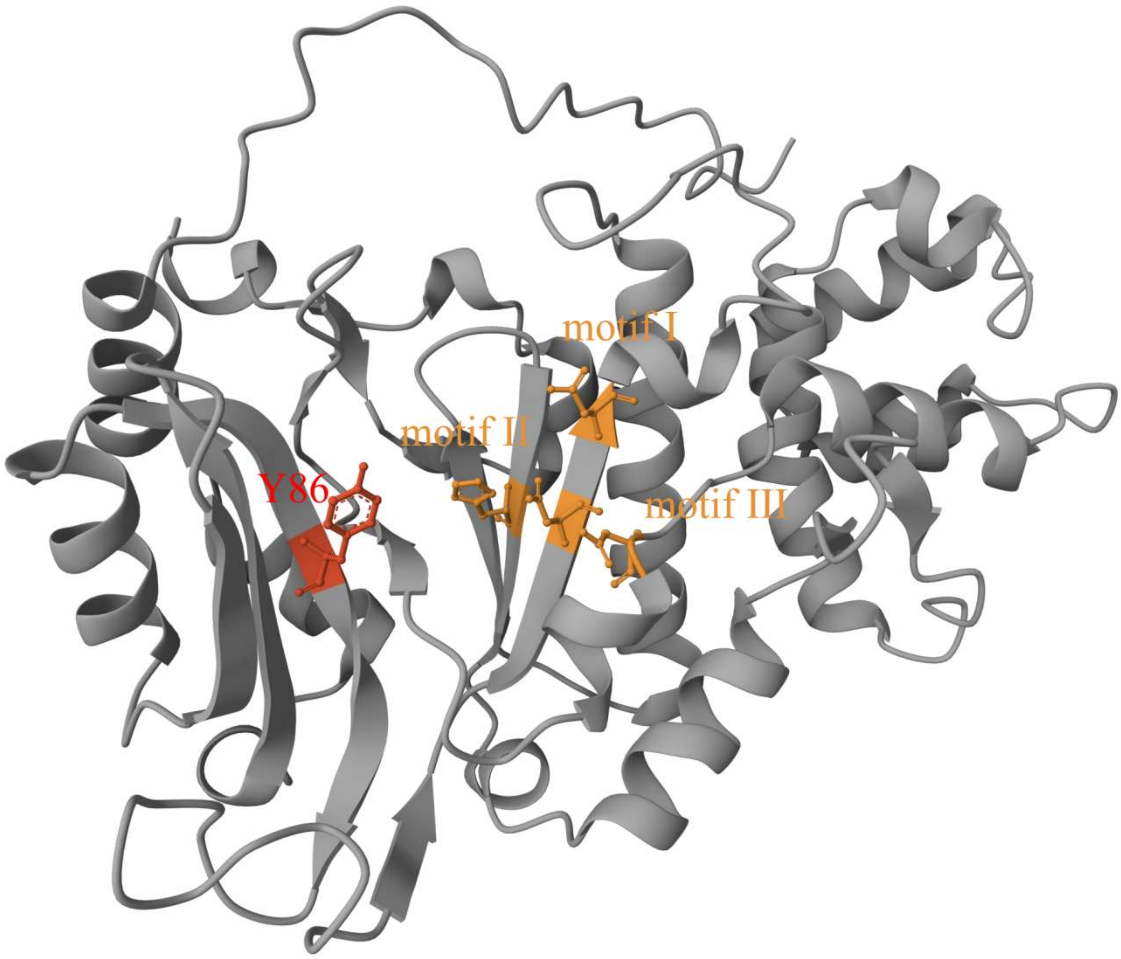

Supplementary Figure 9. Schematic representation of the structure of Gerd_ePriS. Motifs I, II, and III, the three conserved motifs in the AEP superfamily, are labeled with the conserved amino acid residues highlighted. Y86 is the first amino acid residue of motif G/S in Gerd_PriS, the site where point mutations were performed in this study. Structural visualization was performed by MolStar on RCSB PDB.

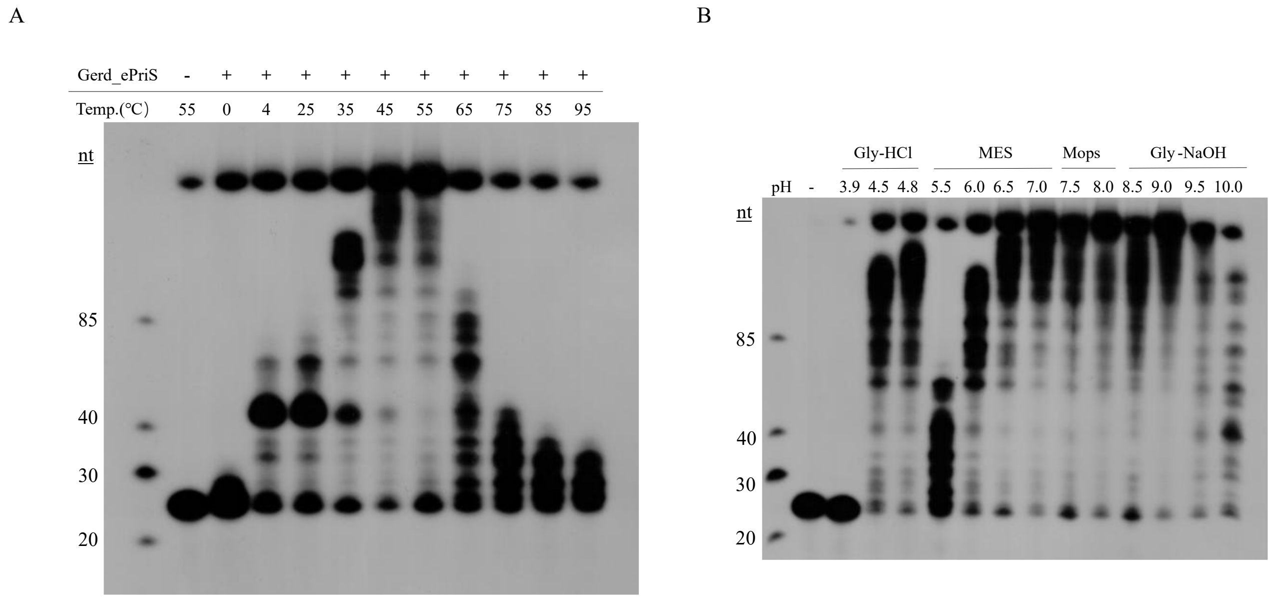

Supplementary Figure 10. Effect of temperature and pH on primer extension by B18_G1 primase. (A) Temperature. The reaction mixture, containing Gerd_ePriS (1.5 μM), 4 nM ^{32}P-labeled D25 primer annealed to M13mp18 ssDNA, 10 μM dNTPs, 50 mM MES-NaOH, pH7.0, 100 μg/ml BSA, and 10 mM $MnCl_2$, was incubated for 30 min at indicated temperatures. **(B)** pH. Reactions were performed for 30 min at 55°C in the same mixture as in **(A)**, except for the substitution of Gerd_ePriSL for Gerd_ePriS, and in indicated buffers, instead of MES-NaOH. Reactions were stopped by the addition of SDS (0.8%) and protease K (1.6 mg/ml), and then analyzed on 15% polyacrylamide gel (19:1) containing 8 M urea. The gel was exposed to X-ray film.

Supplementary table 1. Strains and oligonucleotides used in this study.

**species or fragment name**	**statement**	**sequences**
Pyrococcus furiosus	Genome DNA (Kept in our lab)	–
Sulfolobus solfataricus P2	Strain and Genome DNA (Kept in our lab)	–
dT35	ssDNA	5’-TTTTTTTTTTTTTTTTTTTTTTTTTTTTTTTTTTT
SP2	ssDNA	5’TTTTTTTTTTTTTTTGTGTCGCAGCTGCCACCCTTTTT TTTTT
D25	ssDNA	5'-GTACCGAGCTCGAATTCGTAATCAT
R25	ssRNA	5'-GUACCGAGCUCGAAUUCGUAAUCAU

Supplementary table 2. The database IDs of the structures used in this study.

**Protein**	**ID**	**database**
PriS		
Gerd_PriS	A0A497RBE9	AlphaFoldDB
Homo sapiens PriS	4MHQ	PDB
Prometheoarchaeum syntrophicum PriS	A0A5B9DFU6	AlphaFoldDB
Pyrococcus furiosus PriS	1G71	PDB
Saccharolobus solfataricus PriS	5OF	PDB
PriL		
Gerd_ePriL	A0A497R849	AlphaFoldDB
Gerd_aPriL1	A0A497QTN0	AlphaFoldDB
Gerd_aPriL2	A0A497QRQ5	AlphaFoldDB
Homo sapiens PriS	P49643	AlphaFoldDB
Prometheoarchaeum syntrophicum PriL	A0A5B9DG3	AlphaFoldDB
Pyrococcus furiosus PriL	Q8U4H7	AlphaFoldDB
Saccharolobus solfataricus PriL	Q9UWW1	AlphaFoldDB

Supplementary table 3. Structure-resolved and -unresolved portions of PriL.

species (taxonomy)	PDB ID	resolved part	unresolved part
Saccharolobus solfataricus	5OF3	3-266 aa & 293-306aa	267-292 aa
Pyrococcus abyssi	9F28	1-211 aa	212-393 aa
Pyrococcus horikoshii	2DLA	1-222 aa	213-394 aa
Homo sapiens	5EXR	22-455 aa	456-509 aa

Supplementary table 4. Information on structural alignment between Gerd_ePriS and PriS from other organisms.

Entry	RMSD	TM-score	Identity	Aligned Residues	Sequence Length	Modeled Residues
Gerd_PriS	-	-	-	-	344	344
Promethearchaeum syntrophicum PriS	4.03	0.62	20%	218	378	378
Homo sapiens PriS	3.09	0.81	30%	284	425	400
Pyrococcus furiosus PriS	3.86	0.74	20%	255	347	344
Saccharolobus solfataricus PriS	2.66	0.62	25%	222	330	319

RMSD: Root Mean Square Deviation

Supplementary table 5. Information on structural alignment between N-terminal portions of Gerd_ePriL and PriL from other organisms.

Entry	RMSD	TM-score	Identity	Aligned Residues	Sequence Length	Modeled Residue
Gerd_ePriL	-	-	-	-	444	240
Gerd_aPriL1	3.11	0.66	12%	172	350	230
Gerd_aPriL2	3.21	0.67	13%	174	340	220
Homo sapiens PriL	3.52	0.7	14%	181	509	260
Pyrococcus furiosus PriL	3.4	0.67	12%	179	396	220
Promethearchaeum syntrophicum PriL	3.64	0.66	7%	166	419	250
Saccharolobus solfataricus PriL	3.44	0.62	15%	166	307	210

Supplementary table 6. Information on structural alignment information between C-terminal domains of Gerd_ePriL and PriL from other organisms.

Entry	RMSD	TM-score	Identity	Aligned Residues	Sequence Length	Modeled Residue
Gerd_ePriL	-	-	-	-	444	204
Gerd_aPriL1	2.25	0.51	27%	113	350	120
Gerd_aPriL2	2.14	0.51	26%	114	340	120
Homo sapiens PriL	2.37	0.77	27%	164	509	250
Pyrococcus furiosus PriL	3.29	0.48	21%	107	396	176
Promethearchaeum syntrophicum PriL	3.24	0.54	24%	117	419	169
Saccharolobus solfataricus PriL	2.11	0.42	17%	94	307	97
